# Current trends in pharmaceutical industry: Post -CoVid-19 pandemic effects

**DOI:** 10.6026/9732063002001784

**Published:** 2024-12-31

**Authors:** Monisha Mohan, Kalaiselvi Rajendiran, Yuvhraj Rajaram, Shreya Balaji, Anandavayaravel Cassinadane

**Affiliations:** 1 Department of Biochemistry, Trichy SRM Medical College Hospital and research centre, Irungalur, Trichy, India; 2 Department of Biochemistry, Panimalar Medical College Hospital & Research Institute, Chennai, India; 3 Department of Pharmacy, Shri Venkateswara College of Pharmacy, Ariyur, Puducherry, India; 4 Department of Biomedical Science, Sri Ramachandra Institute of Higher Education and Research, Porur, Chennai, India; 5 Department of Paramedical Sciences, Sri Venkateswara College of Paramedical Sciences, Ariyur, Puducherry, India

**Keywords:** Artificial intelligence, pharmaceutical, block chain technology

## Abstract

The pharmaceutical industry is undergoing a period of significant transformation. This is driven by advances in technology, increased regulatory scrutiny and evolving market demands. The post Covid-19 pandemic era has affected the pharmaceutical industry to meet these requirements. In this regard, emerging trends include the adoption of digitalization, block chain technology and de-centralized clinical trials. Hence, we review the current trends describing evolving challenges post Covid-19 pandemic.

## Background:

The pharmaceutical industry plays a significant role in providing global healthcare by developing ground-breaking treatments, vaccines and therapies. In recent years, the industry has witnessed significant changes driven by technological advancements, regulatory shifts and evolving patient expectations. The CoVid-19 pandemic, in particular, accelerated trends such as the adoption of digital health technologies and decentralized clinical trials (DCTs), highlighting the need for an adaptable and resilient drug development process [1, 2]. Nevertheless, these advancements are accompanied by a few complex challenges, such as the management of complex supply chains, the navigation of regulatory variations across different markets and the high costs of research and development (R & D) [3, 4-5]. New digital techniques like machine learning and artificial intelligence (AI) have emerged to improve drug discovery, but they also present issues with data protection, integration and interpretation within regulatory frameworks [6]. Therefore, it is of interest to describe the challenges faced post-CoVid-19 pandemic in the pharmaceutical industry.

## Post-pandemic challenges:

## Increased demand:

The pharmaceutical industries have been tumbled by the CoVid-19 pandemic of its rapid spread and indefinite treatment. The increased need for pharmaceuticals have been moved towards research in developing treatment strategies and drug supply during the crisis time to meet the increased demand [7]. The overspread of the pandemic, overbuying due to panic and supply chain shortage have increased the demand of medicines. Panic buying and stocking of drugs for the treatment of chronic ailments by the general public was reported worldwide [8, 9]. There was a significant increase in the demand for medications treating chronic conditions such as hypertension, diabetes and mental health disorders. It has been reported that countries like Australia and Germany effectively managed this surge through stock regulation and supply-demand balancing [10]. However, the global pharmaceutical industry faced significant obstacles with the disrupted supply of active pharmaceutical ingredients (APIs) from the primary suppliers, China and India. This commotion, coupled with increased demand, led to price hikes for essential drugs such as amoxicillin, ceftriaxone and ciprofloxacin [10, 11]. The other common drugs added to the demand list by the Food and Drug Association (FDA) were the covid related medications like hydroxyl-chloroquine, chloroquine, azithromycin, dopamine, dobutamine, fentanyl, heparin, midazolam, propofol, dexmedetomidine [12]. Increased demand for drugs from 100% to 700% was noted for respiratory treatments, sedatives and pain treatments. This is due to the disruption in the supply and transport of raw materials required for production [7]. Increased demand created an increase in price of the essential drugs during the pandemic. The cost increase was reflected by antibiotics like amoxicillin, potassium clavulanate, ceftriaxone potassium sterile, meropenam, vancomycin, gentamycin and ciprofloxacin. Therefore, the world's demand for pharmaceuticals dominated the domestic consumption of the drugs which was restricted by the Indian Pharmaceutical Alliance (IPA). As a result, drug demand affected the Indian trade and was reported to be about 10-15% which reached 50% for some pharmaceuticals [7].

## Supply chain deficiency:

Long before the pandemic, a coordinated approach to reporting medicine issues and shortages was set up, which reported the risks of supply chain vulnerabilities [13]. During the pandemic, supply chain shortages increased as hospitalizations led to shortages of both CoVid-19 and non - CoVid-19 medications. Workforce shortages and transportation disruptions further strained the supply chain, particularly in countries like India, where lockdowns severely impacted production and distribution capabilities [10].

## Shifts to telehealth:

The pandemic accelerated the adoption of telehealth and telepharmacy services. These technologies became essential for maintaining healthcare delivery while minimizing face-to-face interactions. However, this shift posed challenges, particularly for older adults who may lack access to or familiarity with digital tools [14]. The rapid transition to telehealth underscored the need for enhanced digital literacy and access to ensure equitable healthcare delivery. Face-to-face interactions were shifted to telecommunication services, which reduced the utility of health services due to substandard knowledge to the use of software and no access to this facility by the older generation, as they are the common consumers and customers of drug industry [15].

## Demands for research and development:

Disease related database collection and researches have guided the policy makers in effective planning to tackle the morbidity state [16]. Policy makers directly relied on the R & D to provide effective control of the spread of CoVid and the vaccine to overcome morbidity. Collaboration by different R & D for CoVid-19 vaccine production was initiated worldwide [17]. Conducting clinical trials for vaccine was time-consuming and complex with multiple process requirements like regulatory and ethical approvals, site qualification and cold chain approval. Several post-approval challenges have been faced in the post pandemic times which are listed below [18].

## Logistical challenges:

[1] Travel restrictions during pandemic (lack of public transport)

[2] Monitors cannot travel to sites

[3] Patients cannot travel to sites easily

## Resource challenges:

[1] Cold chain requirement for mRNA vaccines resulted in

[2] Shortage of freezers for other clinical studies

[3] Mass vaccinations and CoVid testing put a strain on laboratory resources

[4] Sick site personnel resulted in loss of precious time to conduct trials

[5] Reduced patient traffic to doctor offices resulting in difficulties in enrollment for trials

## Management of clinical trials:

During the clinical trials, data fragmentation and disconnected system involvement arises from dispersed data which has to be analyzed. This extensive manual data transcription, are some of the major challenges associated with traditional clinical trials. The trial models have not been inventive enough, which necessitated redesign and repetition of the current work. Easier approach to enrolment, monitoring, retention and medical adherence was mandated. Another major challenge faced is the trial participant's time-consuming travel process to the trial locations. It has an impact on patient enrolment and patient re-registration in the same setting is facilitated by frequent site visits [19, 20].

## Developing trend:

Digitalization has brought extensive advancements from research and development (R & D) to supply chain, manufacturing, regulatory compliance and patient engagement. The incorporation of latest digital technologies such as AI, block chain technology, telemedicine and DCT are the trends that came into existence in the post-pandemic era [1, 22]. Computerization, automation and robotics have enabled cost reduction, increased production and efficiency and adaptability [23]. Added to these, patent expirations, increasing customer demand, rising competition and rising pricing pressures challenge the pharmaceutical industry. Drogana *et al.* have explained that scientific digitisation improves collaboration and product quality by streamlining operations, reducing errors and enhancing efficiency to adapt to the economy and boost productivity. Training and up-skilling in digitalisation can have a significant impact on productivity. True digitalization should encompass all businesses and industries, with small and mid-sized businesses adopting technology and large corporations grappling with technological challenges [24]. Investment in digital transformation could lead to increased revenue growth and return on investment, ultimately enhancing drug productivity [25]. Therefore, Digital health during the CoVid-19 pandemic has evolved very rapidly, with different applications and roles aimed at curbing the pandemic [26].

In pharmaceutics, AI technology spans various divisions like drug discovery, precision medicine, formulation optimization, clinical trials, safety monitoring and supply chain management [26]. Formulations are customized for the demands of each patient through the logical design of intelligent drug carriers, such as AI-optimized liposomes for cancer treatment. AI-powered sensors, such as glucose-monitoring biosensors for diabetics, allow for adaptive medication delivery, improving accuracy [27]. AI-powered predictive algorithms have been applied in appropriate patient groups to expedite the hiring process and to track the status of trials in real time [28]. In drug design, AI models have been used to identify the correlations between a compound's chemical structure and its biological activity [29]. AI has introduced several method domains, such as reasoning, knowledge representation and solution search, among them, a basic concept of machine learning (ML). ML uses algorithms where a set of data is analyzed by recognized patterns. A subfield of the ML is deep learning (DL), which includes artificial neural networks (ANNs) [30]. These aids therapeutic target identification by analyzing a variety of data sources, including clinical, proteomic and genomic data. Also helps development of drugs that can alter biological processes by identifying targets and molecular pathways linked to diseases [31]. It also facilitates the screening of chemical libraries to find drug candidates with the probability of binding to a specific target [32]. Thus, saves time and money for researchers by prioritizing and choosing compounds for experimental testing by modeling chemical interactions and predicting binding affinities [33]. Qureshi *et al.* have shown that reinforcement learning and generative models have been used by AI algorithms to suggest new chemical compounds that resemble drugs. AI has broadened the chemical space and supports the creation of novel treatment options by drawing knowledge from chemical libraries and experimental data [34]. In the case of repurposing drugs, AI has been employed to analyze the existing drugs used for treating another conditions for a new purposes. This approach adds to time saving and cost reduction of drug research. The traditional trials are overcome with AI which could assist in both optimization and accumulation of work associated with developing a patient-centric design [26].

## Block chain technology:

Supply chain disruptions have been disturbed by pandemics, natural disasters, price adjustments, cyber-attacks, delays in logistics and problems with products. The epidemic's effects on transportation have decimated international enterprises and the supply chain. For compliance and patient demands, footprint adjustments must be manufactured. Due to issues with maintaining the cold chain, a sizable number of COVID-19 vaccinations from the pharmaceutical sector were rendered useless during the pandemic [35].

To overcome the supply chain disruptions, BT, newer technology evolved in the last decade that have brought an end-to-end visibility in the supply chain, allowing stakeholders to track drugs from production to end-user delivery. Block chains are generally unbreakable shared database which employs networks of peers to store blocks of data for cryptographically bonded operations. By using unique identifiers, companies can verify the authenticity of each drug, preventing the circulation of counterfeit medicines. The fundamental characteristics of BT are transparency, decentralization and immutability. This promised a stable situation that improved supply chain security and efficiency [36, 37]. Nowadays, BT in pharma industry is recognized to be a "pharma-chain" that acts as decentralized hyper-ledger fabric framework to take care of interoperability, accountability and secrecy. Interoperability is one such important arena to be taken care post pandemic time. BT provided a building blocks for the trading partners and consumers where/the transparency of trusted and secured data is being used and to synchronize processes through a mutually agreed rule set. This has become compatible enough with supply chain disruption during pandemic [38]. Online surveys have shown that most customers viewed this new BT optimistically for the medical supply chain [39]. BT allows for smart contracts to establish data provenance and allows for both off-chain and on-chain storage for safe, quick transactions [40].

## Decentralized clinical trials (DCT):

The COVID-19 pandemic underscored the necessity of rapid vaccine and drug development, leading to the widespread adoption of DCTs [41]. DCTs are the type of clinical trial conducted remotely, outside of a traditional clinical setting. DCTs offer a more convenient, cost-effective and patient-centered approach to conducting clinical trials, allowing patients to participate from home and contribute to medical research advancements [42]. The DCT model has been reported to reduce dependency on centralized trial sites by incorporating remote data collection methods. This approach is done by remote intervention, social media recruitment, study task reminders, e-consent, participant-informed study design and the return of results for participants. This way provides patient convenience and reduces dropout rates, as patients can engage in studies from the comfort of their homes [43]. Therefore, DCTs have turned into patient-centered, efficient alternative to conventional trial designs, with potential to facilitate future clinical research. Tools such as electronic health records, telemedicine, wearable health devices and remote monitoring are known to show improved trial accessibility and efficiency [44]. By incorporating telemedicine, DCT has been facilitated by consultations and follow-ups to be conducted remotely, facilitating seamless trial participation and data collection. Additionally, DCTs support faster data analysis with improved digital monitoring tools and real-time data transmission [45, 46]. DCT also has been shown to promote diversity in clinical research by accommodating different ethnicity-based participants and has been reported that efforts must be made to build trust among marginalized communities [43].

Concerning drug stability, storage and unauthorized access in DCT, the shipment of drugs directly to patient's homes played a central key role in DCT; the drugs were stored under appropriate conditions to maintain their stability and effectiveness. Temperature-sensitive drugs, in particular, necessitate advanced storage solutions and real-time temperature monitoring to ensure proper storage throughout the supply chain. Security measures were a key feature to prevent unauthorized access to medications. Innovations such as biometric authentication, secure storage containers and tamper-evident packaging have been employed to ensure that only authorized patients will be able to access the drugs. These security features not only protect drug integrity but also enhance patient confidence in the trial process. The other trend was the patient communication system and refill process in DCT, where the communication system between patients, storage systems and drug suppliers to prevent interruptions in treatment was critically a challenging role in clinical trials. Automated systems and refill reminders have been used to streamline the refill process and facilitate timely communication between the storage and distribution centers, ensuring uninterrupted access to medication [42]. Regulatory compliance has been a challenging one in DCT, where the Local laws and regulations around drug dispensing vary by region, posed a significant challenge for DCTs that operate across multiple jurisdictions. Compliance with these diverse regulatory requirements is essential for ethical and legal trial conduct. Regulators have identified many major issues with DCTs, including participant safety and investigator monitoring when there are few in-person interactions and physical examinations [47]. Staying informed and proactively addressing regulatory variations helped trial sponsors to mitigate legal risks and maintain trial integrity [42].

## Conclusion:

The development and applications of artificial intelligence, block chain technology and decentralized clinical trials has evolved during the post pandemic era in the pharmaceutical industry. This will help to uphold the challenges like drug demand, design, discovery and delivery.

## References

[1] Getachew E (2023). Front Public Health..

[2] https://www.fda.gov/drugs/cder-conversations/evolving-role-decentralized-clinical-trials-and-digital-health-technologies.

[3] simoesns S, Hius I (2021). Front Med (Lausanne)..

[4] Socal M.P (2021). Am J Public Health..

[5] https://www.cbo.gov/publication/57126.

[6] Topol E.J (2019). Nat Med..

[7] Ayati N (2020). Daru..

[8] Shukar S (2021). Front Pharmacol..

[9] Kostev K, Lauterbach S (2020). J Psychiatr Res..

[10] https://pharmaceutical-journal.com/article/research/responding-to-disruptions-in-the-pharmaceutical-supply-chain.

[11] Socal M.P (2021). Am J Public Health..

[12] https://dps.fda.gov/drugshortages.

[13] https://iris.who.int/bitstream/handle/10665/331028/DI302-180-185-eng.pdf.

[14] https://www.mckinsey.com/industries/life-sciences/our-insights/a-new-operating-model-for-pharma-how-the-pandemic-has-influenced-priorities.

[15] Choi N.G (2022). J Appl Gerontol..

[16] https://cbhidghs.mohfw.gov.in/WriteReadData/l892s/94203846761680514146.pdf.

[17] Druedahl L.C (2021). Vaccine..

[18] Shojaei A, Salari P (2020). DARU..

[19] https://www.regulations.gov/docket/FDA-2019-D-1264.

[20] Sampson R (2022). J Clin Transl Sci..

[21] Tagde P (2021). Environ Sci Pollut Res Int..

[22] Vayena E (2023). Lancet Digit Health.

[23] Hole G (2021). Int J Pharm X..

[24] Dragana R, Sasa P (2023). Technological Forecasting and Social Change..

[25] Vora L.K (2023). Pharmaceutics..

[26] Alghamdi N.S, Alghamdi S.M (2022). Int J Environ Res Public Health..

[27] Aundhia C (2024). Curr Top Med Chem..

[28] Xiaoran Lu (2024). Journal of the American Medical Informatics Association.

[29] Ovhal Y (2024). International Journal of Pharmaceutical Sciences..

[30] Sarker I.H (2021). N Comput Sci..

[31] Visan A.I, Negut I (2024). Life (Basel)..

[32] Gentile F (2022). Nat Protoc..

[33] Baum Z.J (2021). J ChemInf Model..

[34] Qureshi R (2023). Heliyon..

[35] Lemos F (2024). Thunderbird Int. Bus. Rev..

[36] Wasim Akram (2024). Research in Social and Administrative Pharmacy..

[37] Marko Holbl (2018). Symmetry..

[38] https://www.weforum.org/stories/2020/06/this-is-how-blockchain-can-be-used-in-supply-chains-to-shape-a-post-covid-19-economic-recovery/.

[39] https://tore.tuhh.de/entities/publication/135b235d-5f9a-4b78-a5ae-332a56954b73.

[40] https://ieeexplore.ieee.org/document/10076939.

[41] Suman A (2022). Trials..

[42] Van Norman G.A (2021). JACC Basic Transl Sci..

[43] Hanley-Jr D.F (2023). J Clin Transl Sci..

[44] https://www.fda.gov/drugs/cder-conversations/evolving-role-decentralized-clinical-trials-and-digital-health-technologies.

[45] Peyser N.D (2022). Contemp Clin Trials..

[46] Copland R.R (2024). J Med Internet Res..

[47] De Jong A.J (2022). Clin Pharmacol Ther..

